# Evaluation of the Effect of Thickeners in Enteral Formulas on the Gastric Emptying Rate of Proteins and Carbohydrates Using a Semi-Dynamic Gastric Model

**DOI:** 10.3390/nu16132115

**Published:** 2024-07-02

**Authors:** Motoki Tsukiashi, Takahiro Koyama, Hiroshi Iwamoto, Hirofumi Sonoki, Kazuhiro Miyaji

**Affiliations:** 1 Health Care & Nutritional Science Institute, R&D Division, Morinaga Milk Industry Co., Ltd., 5-1-83 Higashihara, Zama 252-8583, Kanagawa, Japan; 2 Health Science Research Center, R&D Institute, Morinaga & Co., Ltd., 2-1-1 Shimosueyoshi, Tsurumi-ku, Yokohama 230-8504, Kanagawa, Japan

**Keywords:** enteral formula, gastric emptying, semi-dynamic gastric model, agar, carrageenan, aggregation, protein

## Abstract

The emptying rate of specific nutrients in enteral formulas is poorly understood, despite the importance of controlling the emptying rate in tube-fed patients. Because of their viscosity, thickened formulas are widely used to avoid gastric reflux and reduce the burden on caregivers. This study examined how thickeners in enteral formulas affected the gastric emptying rates of proteins and carbohydrates. A semi-dynamic gastric model was used to prepare and digest test enteral formulas that contained either no thickeners or agar (0.2%). The amounts of protein and carbohydrates in each emptied aliquot were determined, and the emptying rate was calculated. We found that agar accelerated protein emptying, and an exploratory experiment with agar (0.5%) suggested the possibility of concentration dependence. Additionally, experiments using gellan gum (0.08%), guar gum (0.2%), or carrageenan (0.08%, 0.2%) suggested that protein emptying could vary depending on the thickener type and that carrageenan might slow it. These results could help with the appropriate selection of thickeners added to liquid foods based on the patient’s metabolic profile to manage nutrition, not only for tube-fed patients but also for those with oropharyngeal dysphagia or diabetes.

## 1. Introduction

Enteral nutrition (EN) provides essential nutrients to patients who cannot consume sufficient food orally to meet their nutritional requirements [1]. The EN formula is typically given slowly over a long period of time to prevent diarrhea caused by rapid gastric emptying and gastroesophageal reflux. However, prolonged administration of enteral formulas reduces the patient’s quality of life and increases the workload for caregivers. To address these problems, the practice of increasing the viscosity of enteral formulas has gradually gained popularity [2,3,4,5]. Increasing food viscosity using thickeners is a common procedure in nursing care. Thickened beverages and soup, for example, have long been used for patients with oropharyngeal dysphagia [6,7,8]. Mixtures of agar and liquid enteral formula, thickened formulas (a few thousand mPa·s), and semi-solid enteral formulas (10,000~20,000 mPa·s) help to prevent gastroesophageal reflux because of their viscosity [2,3,4,5,9], thereby enabling a reduction in administration time [2,3,5,10], leading to a decrease in the burden on both patients and caregivers.

Controlling the rate of nutrient digestion and absorption based on the patient’s metabolic constitution requires the proper management of the gastric emptying rate of each nutrient. Muscle atrophy and weakness, which are associated with clinical prognosis [11], are common in tube-fed patients. Promoting anabolism through correct amino acid absorption kinetics can alleviate this problem [12]. Therefore, regulating the gastric emptying rate of proteins in tube-fed patients is important.

Hyperglycemia is another common problem in tube-fed patients and is associated with mortality [13]. Reducing the carbohydrate gastric emptying rate while increasing that of proteins, which are known to stimulate incretin secretion [14], could help manage postprandial blood glucose levels.

Few studies have investigated the nutritional benefits of viscosity-adjusted enteral formulas. In one of the few studies, it was found that a semi-solid enteral formula increases stomach retention time and prevents postprandial blood glucose elevation [15]. However, whether the thickened formula helps to lower blood glucose levels remains unclear. To our knowledge, no research has examined the effect of the type of thickener and its impact on the gastric emptying of other nutritional components, such as proteins. Although Markussen et al. found that the formation of milk protein aggregates in the stomach varies depending on the thickener used [16], the effects of thickeners on more complex diets, such as enteral formulas, are unclear. Despite the necessity of managing the gastric emptying rate of each nutrient individually in tube-fed patients, the influence of thickeners on the gastric emptying rate of each nutrient in enteral formulas is unclear.

The semi-dynamic gastric model is a standardized in vitro digestion method that can perform both physical and chemical digestion over time and analyze the variations in the gastric emptying rate of each nutrient separately [17]. Mulet-Cabero et al. used a semi-dynamic gastric model to demonstrate how milk sterilization and homogenization alter digestion dynamics [18].

In this study, we investigated how thickeners affected the gastric emptying rate of each nutrient. Therefore, we produced thickened formulas using agar, which is a commonly used thickener, and the gastric emptying rates of proteins and carbohydrates were measured using the semi-dynamic gastric model. Furthermore, we also conducted exploratory investigations on three other types of thickeners and examined the effects of these types of thickeners.

## 2. Materials and Methods

### 2.1. Materials

All chemicals were of standard analytical grade. KH_2_PO_4_, 6 M HCl, and 4 M NaOH were acquired from FUJIFILM Wako Pure Chemical Corporation (Osaka, Japan). Kanto Chemical Co., Inc. (Tokyo, Japan) supplied the phenol and the (NH_4_)_2_PO_4_. L-aspartic acid was obtained from Kishida Chemical Co., Ltd. (Osaka, Japan). Sigma-Aldrich (St. Louis, MO, USA) provided the porcine pepsin and R. oryzae lipase (Cat. P7012 and 80612, respectively). The enzymatic activity of pepsin was determined using the protocol described by Minekus et al. [19], while lipase was determined using product data sheets. All of the thickeners were obtained from San-Ei Gen F.F.I., Inc. (Osaka, Japan). All other chemicals were purchased from Kokusan Chemical Co., Ltd. (Tokyo, Japan).

### 2.2. Preparation of Test Enteral Formulas

In this study, seven enteral formulas were prepared: a control enteral formula without thickener (control), and six test enteral formulas containing 0.2% agar (AGL), 0.5% agar (AGH), 0.08% gellan gum (GL), 0.2% guar gum (GA), 0.08% carrageenan (CAL), and 0.2% carrageenan (CAH), respectively. These enteral formulas were prepared using standard manufacturing methods, including homogenization and retort sterilization. Each test enteral formula had 4% protein, 16.1% carbohydrate, and 2.5% lipid, primarily derived from sodium caseinate, maltodextrin, and vegetable oil, respectively. The viscosity of AGL, GL, and CAL was 1800 ± 1000 mPa·s, comparable to commercially available thickened formulas. AGH and CAH were included to investigate the concentration dependence of the thickeners. Guar gum had a viscosity below 50 mPa·s. Therefore, the concentration of guar gum in GA was adjusted to 0.2%, corresponding to the concentration of other test enteral formulas. Viscosity was measured using a B-type viscometer (Toki Sangyo Co., Ltd., Tokyo, Japan) at 6 rpm for 120 s at 20 °C.

### 2.3. Semi-Dynamic Gastric Digestion

A semi-dynamic gastric model was developed as previously reported [17]. The oral phase was omitted from this trial because enteral formulas were used. Briefly, 3 mL of simulated gastric fluid (SGF), which was 10% of the total SGF volume, was added to a thermostatically controlled vessel (Metrohm AG, Herisau, Switzerland) at 37 °C. The reaction was subsequently initiated by adding 30 mL of the enteral formula to the vessel. The remaining 90% of the SGF was then added during the reaction using a syringe pump. To avoid enzymatic autolysis, the enzyme solution and other solutions were added separately at constant rates of 10 and 90 µL/min. The reaction time was set at 270 min, based on the calorie density of the test enteral formulas. The vessel was agitated at 35 rpm using a 3D orbital shaker (As One Corporation, Osaka, Japan). Five gastric emptying (GE) points, numbered GE1–GE5, were established by sucking 12 mL of digesta from the bottom of the vessel every 54 min. In other words, GE1, GE2, GE3, GE4, and GE5 represented gastric emptying at 54, 108, 162, 216, and 270 min, respectively. This procedure was performed using an electric pipette (Sartorius, Göttingen, Germany) with a 2 mm tip diameter to simulate the opening diameter of the pylorus [20,21] at a rate of 0.98 mL/s. The pH of the sampled gastric digesta was measured. The enzymatic reaction was then stopped by using 2 M NaOH to raise the pH of the digesta to >7 and boiling it in a water bath for 5 min. The digesta was then freeze-dried using a lyophilizer (Labconco Corporation, Kansas City, MO, USA) and powdered using a grinder (Yasui Kikai Corporation, Osaka, Japan).

### 2.4. Protein Content Analysis

The nitrogen content of the freeze-dried powder was determined using the Dumas method [22] with a SUMIGRAPH^®^ NC-TRINITY (Sumika Chemical Analysis Service, Tokyo, Japan). Protein concentration was calculated by multiplying the nitrogen concentration by 6.38.

### 2.5. Carbohydrate Content Analysis

The carbohydrate concentration of the freeze-dried powder was determined using the phenol–sulfuric acid method [23]. Approximately 30 mg of freeze-dried powder was suspended in 200 mL of Milli-Q water. This suspension (0.5 mL) was blended with 0.5 mL of a 5% phenol solution and mixed thoroughly. Sulfuric acid (2.5 mL) was added to the mixture, which was then thoroughly mixed and allowed to rest for 20 min. The absorbance of the solution was measured at 490 nm using a spectrophotometer (As One Corporation, Osaka, Japan), and the carbohydrate concentration was calculated.

### 2.6. Data Analysis

In this study, the control and AGL were subjected to four independent experiments, and their mean values were presented as the data. In addition, we conducted exploratory investigations on other thickeners and presented the data from only a single experiment.

Statistical analyses were performed using JMP version 13.2.1 (SAS Institute, Cary, NC, USA). The effect at each GE point according to the variety of thickeners during digestion using a semi-dynamic gastric model was evaluated using a Student’s *t*-test. Statistical significance was set at *p* < 0.05.

## 3. Results

### 3.1. Formation of Aggregations during Gastric Digestion

**Figure 1:**
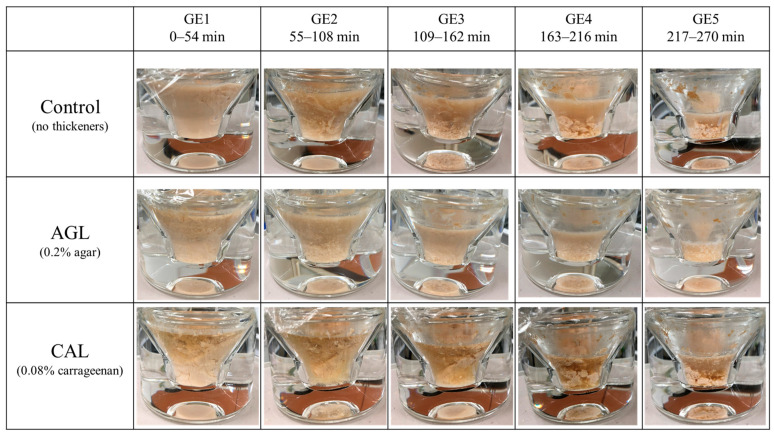
Representative images of the control (no thickeners), AGL (0.2% agar), and CAL (0.08% carrageenan) at each gastric emptying (GE) point during digestion using a semi-dynamic gastric model.

The control formed an aggregation at GE2 that could not be aspirated with a pipette and which lasted until GE5. In contrast, the AGL formed smaller and softer aggregates than those of the control, which could be aspirated using a pipette. Furthermore, AGH produced smaller and softer aggregates than those of AGL. CAL produced stronger aggregates than those of the control group. Moreover, CAH produced stronger aggregates than those of CAL. These results indicate that the aggregation caused by agar or carrageenan was concentration-dependent. Both GL and GA formed aggregates that resembled those in the control group. The images of each gastric emptying point in the control, AGL, and CAL groups, all of which had typical characteristics, are shown in Figure 1.

### 3.2. pH Profile

**Figure 2:**
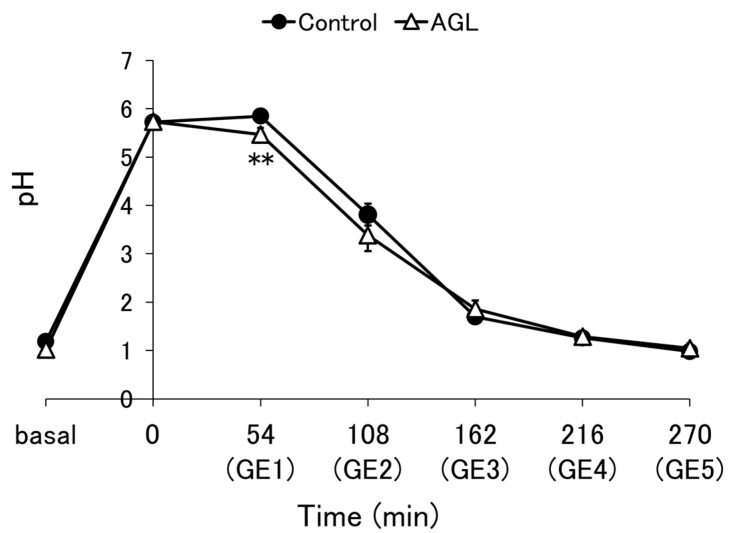
The pH profile is described based on the pH value of the aliquot emptied at each gastric emptying (GE) point during digestion using a semi-dynamic gastric model. Control = enteral formula containing no thickeners; AGL = enteral formula containing 0.2% agar. Data are shown as mean ± SE (*n* = 4). Two asterisks indicate a significant difference at *p* < 0.05 compared to the control (Student’s *t*-test).

**Figure A1:**
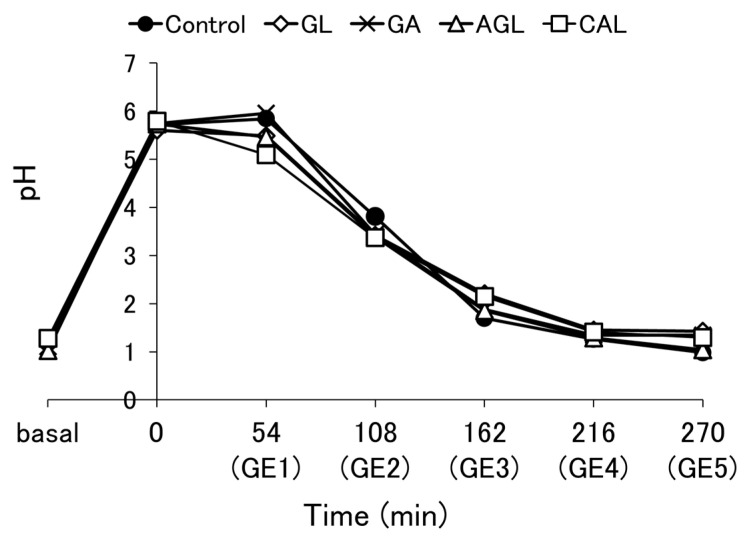
The pH profile is described based on the pH value of the aliquot emptied at each gastric emptying (GE) point according to the variety of thickeners during digestion using a semi-dynamic gastric model. GL = enteral formula containing 0.08% gellan gum; GA = enteral formula containing 0.2% guar gum; and CAL = enteral formula containing 0.08% carrageenan. GL, GA, and CAL each represent data from a single experiment. Control and AGL are shown for reference, using the same data (the mean of four experiments) as presented in Figure 2.

**Figure A2:**
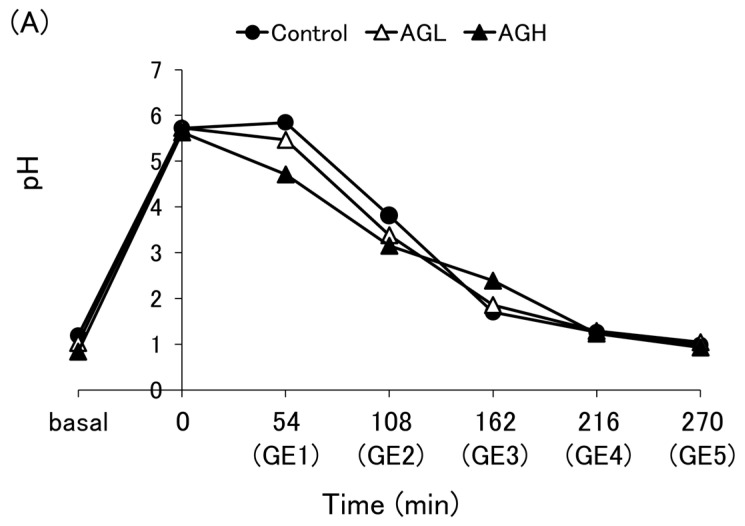

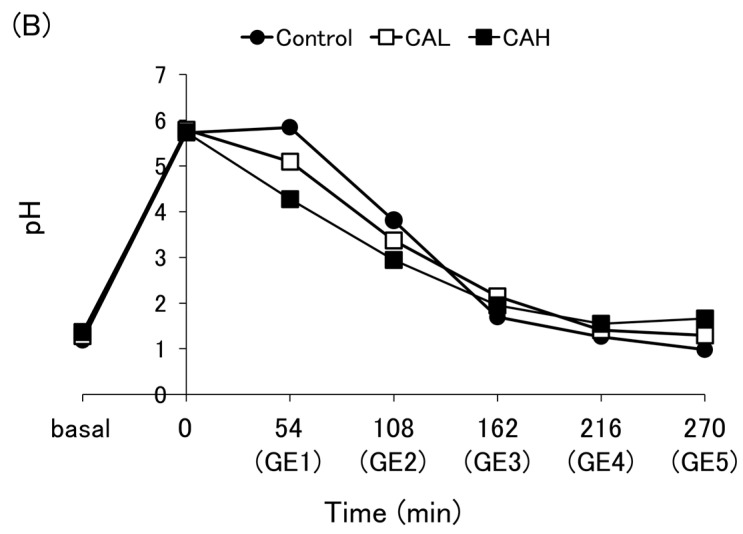
The pH profile is described based on the pH value of the aliquot emptied at each gastric emptying (GE) point according to concentration of (**A**) agar and (**B**) carrageenan during digestion using a semi-dynamic gastric digestion. Control = enteral formula containing no thickeners; AGL = enteral formula containing 0.2% agar; AGH = enteral formula containing 0.5% agar; CAL = enteral formula containing 0.08% carrageenan; and CAH = enteral formula containing 0.2% carrageenan. AGH and CAH each represent the data from a single experiment. Control, AGL, and CAL are shown for reference, using the same data as presented in Figure 2 and Figure A1.

The pH of each aliquot emptied from each enteral formula is shown in Figure 2 and Figure A1, as well as Figure A2A,B. For the control and AGL, the mean of four independent experiments is shown, while the results of a single experiment are shown for the others. Although there was a significant difference in pH between the control and AGL at the GE1 point (*p* = 0.032), the overall tendencies of both were similar (Figure 2). No substantial differences in tendencies were observed for the other thickeners either (Figure A1). The pH increased from approximately 1, simulating basal gastric conditions, to approximately 6, because of the buffering capacity of the enteral formulas. However, because of the inflow of SGF and gastric emptying, the pH then decreased to approximately 1.

### 3.3. Protein Emptying

**Figure 3:**
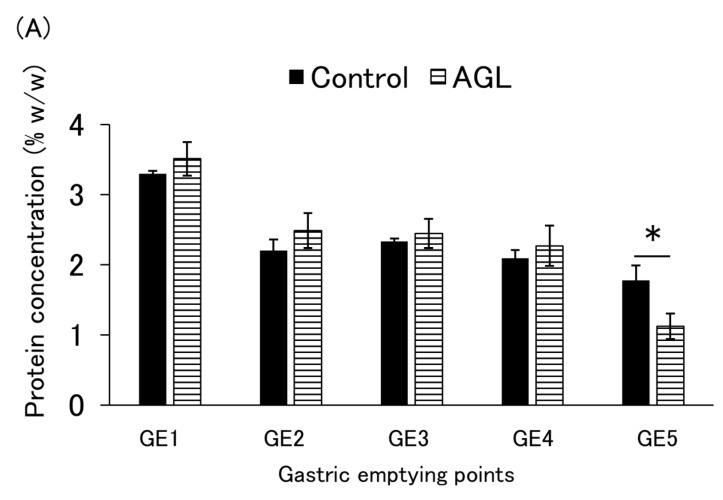

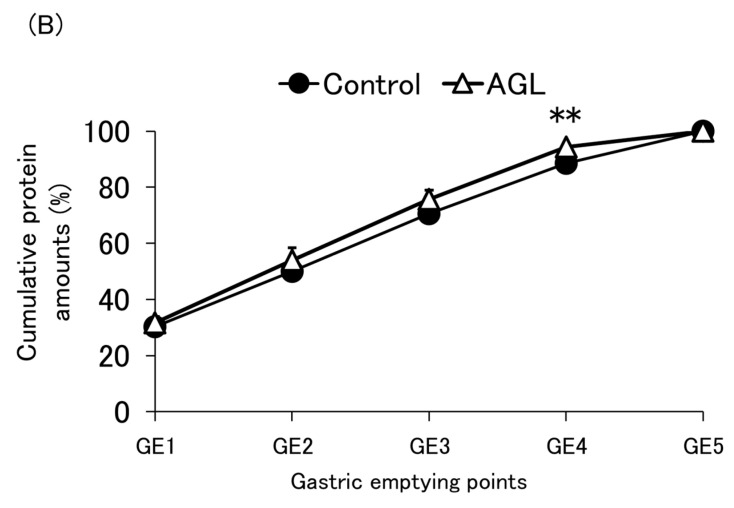
(**A**) Concentration of protein at each gastric emptying (GE) point and (**B**) cumulative protein amounts emptied by the gastric emptying point indicated. Control = enteral formula containing no thickeners; AGL = enteral formula containing 0.2% agar. Data are shown as mean ± SE (*n* = 4). One asterisk indicates a marginally significant difference at *p* < 0.1, and two asterisks indicate a significant difference at *p* < 0.05 compared to the control (Student’s *t*-test).

**Figure A3:**
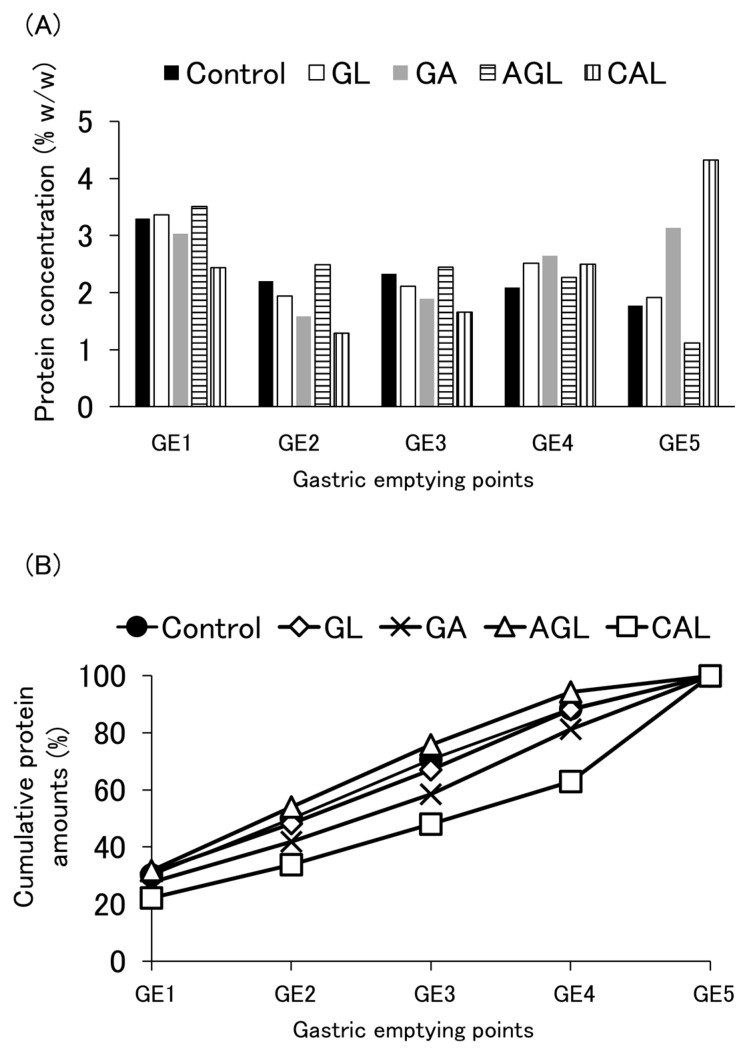
(**A**) Concentration of protein at each gastric emptying (GE) point and (**B**) cumulative protein amounts emptied by the gastric emptying point indicated. GL = enteral formula containing 0.08% gellan gum; GA = enteral formula containing 0.2% guar gum; CAL = enteral formula containing 0.08% carrageenan. GL, GA, and CAL each represent the data from a single experiment. Control and AGL are shown for reference, using the same data (the mean of four experiments) as presented in Figure 3.

**Figure A4:**
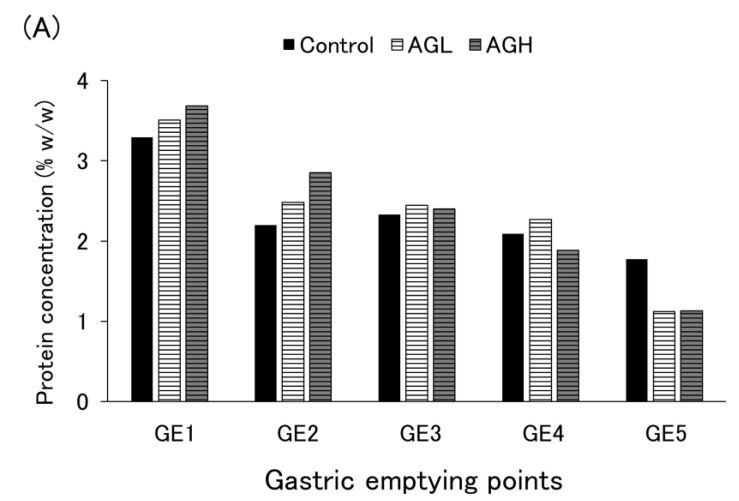

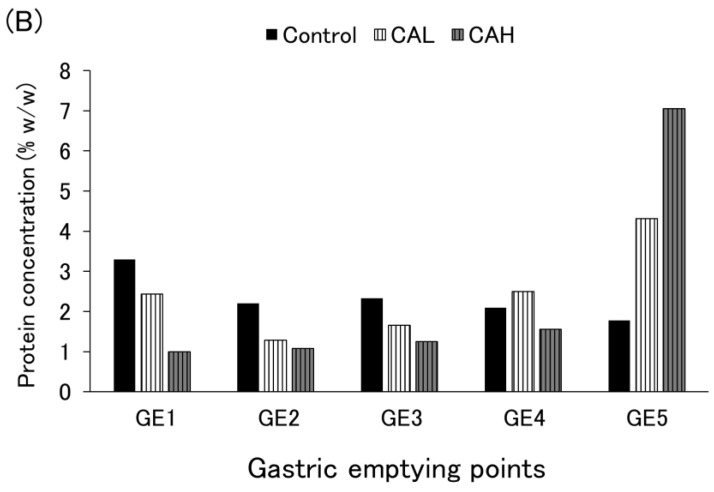
Concentration of protein at each gastric emptying (GE) point according to concentration of (**A**) agar and (**B**) carrageenan during digestion using a semi-dynamic gastric digestion. Control = enteral formula containing no thickeners; AGL = enteral formula containing 0.2% agar; AGH = enteral formula containing 0.5% agar; CAL = enteral formula containing 0.08% carrageenan; and CAH = enteral formula containing 0.2% carrageenan. AGH and CAH each represent the data from a single experiment. Control, AGL, and CAL are shown for reference, using the same data as presented in Figure 3 and Figure A3.

Figure 3A,B and Figure A3A,B show the protein concentration and cumulative protein emptied at each emptying point. For the control and AGL, the mean of four independent experiments is shown, while the results of a single experiment are shown for the others. The AGL protein content decreased proportionally with gastric juice inflow and gastric emptying and showed a trend (*p* = 0.058) to be lower than the control at GE5 (Figure 3A). Consequently, the AGL protein was emptied faster than in the control group and was significantly faster (*p* = 0.018) at GE4 (Figure 3B). Furthermore, this tendency was slightly dependent on the agar concentration (Figure A4A). In contrast, GL and GA showed similar tendencies to the control, with high protein concentrations between GE3 and GE5 (Figure A3A). Interestingly, CAL had higher protein concentrations at GE5 than the control, and the protein in CAL emptied more slowly than that in the control (Figure A3B). Moreover, this tendency was dependent on the carrageenan concentration (Figure A4B).

### 3.4. Carbohydrate Emptying

**Figure 4:**
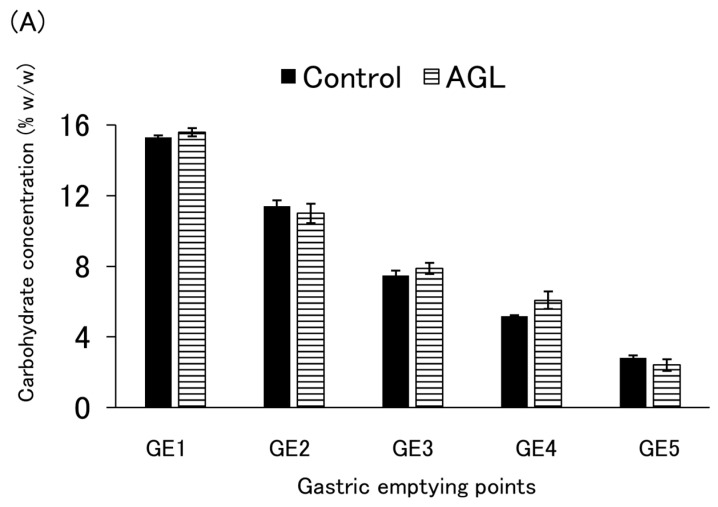

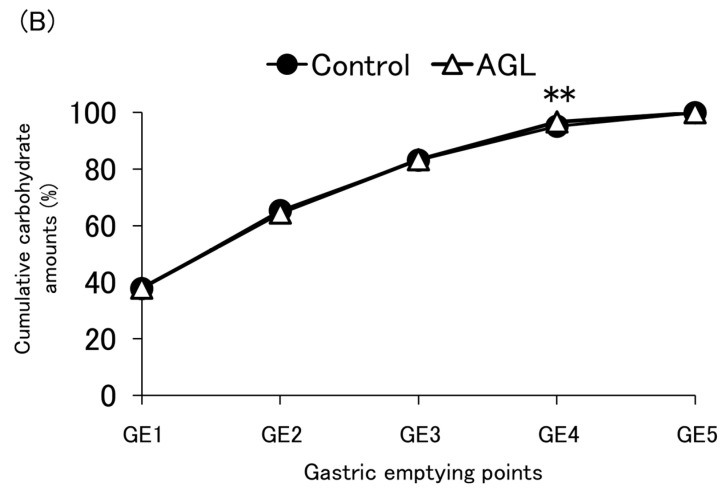
(**A**) Concentration of carbohydrates at each gastric emptying (GE) point and (**B**) cumulative carbohydrate amounts emptied at the gastric emptying point indicated. Control = enteral formula containing no thickeners; AGL = enteral formula containing 0.2% agar. Data are shown as mean ± SE (*n* = 4). Two asterisks indicate a significant difference at *p* < 0.05 compared to the control (Student’s *t*-test).

**Figure A5:**
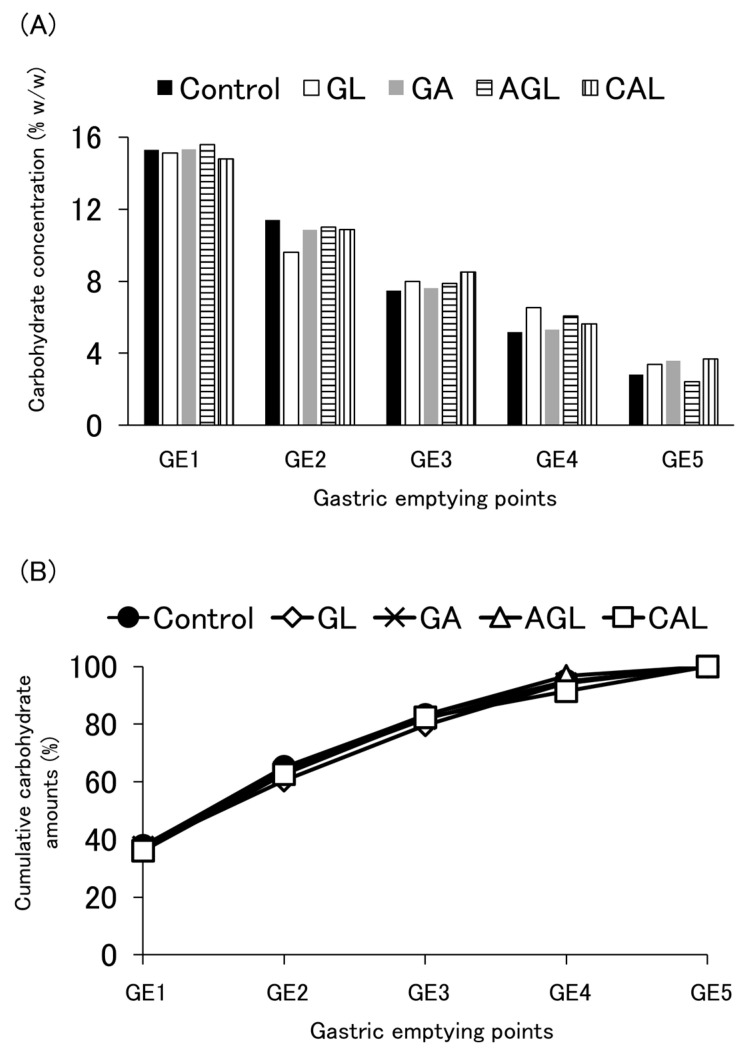
(**A**) Concentration of carbohydrates at each gastric emptying (GE) point and (**B**) cumulative carbohydrate amounts emptied at the gastric emptying point indicated. GL = enteral formula containing 0.08% gellan gum; GA = enteral formula containing 0.2% guar gum; and CAL = enteral formula containing 0.08% carrageenan. GL, GA, and CAL each represent the data from a single experiment. Control and AGL are shown for reference, using the same data (the mean of four experiments) as presented in Figure 4.

**Figure A6:**
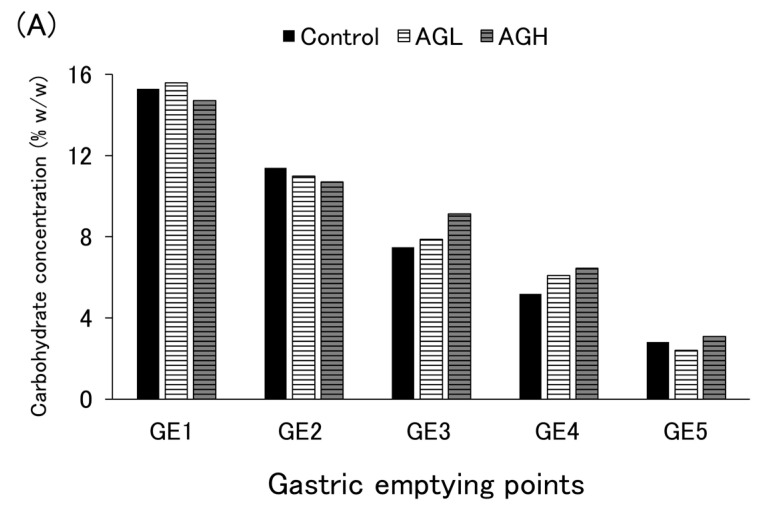

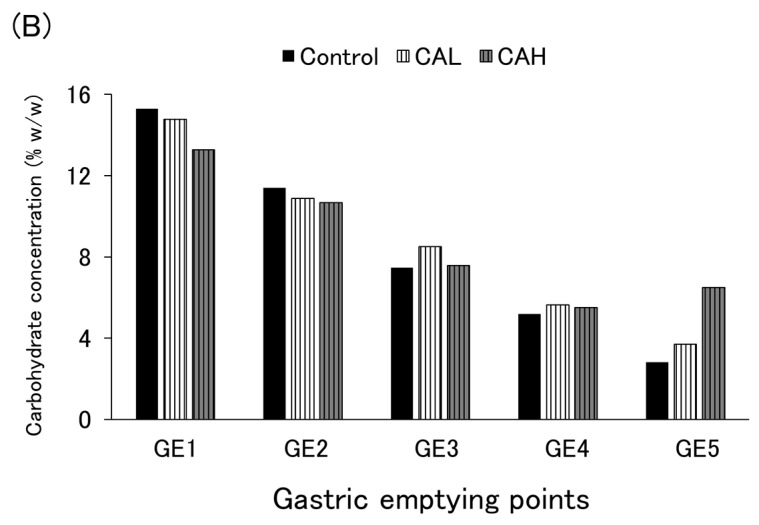
Concentration of carbohydrates at each gastric emptying (GE) point according to concentration of (**A**) agar and (**B**) carrageenan during digestion using a semi-dynamic gastric digestion. Control = enteral formula containing no thickeners; AGL = enteral formula containing 0.2% agar; AGH = enteral formula containing 0.5% agar; CAL = enteral formula containing 0.08% carrageenan; and CAH = enteral formula containing 0.2% carrageenan. AGH and CAH each represent the data from a single experiment. Control, AGL, and CAL are shown for reference, using the same data as presented in Figure 4 and Figure A5.

The carbohydrate concentration and cumulative carbohydrates emptied at each emptying point are shown in Figure 4A,B and Figure A5A,B, as well as Figure A6A,B. For the control and AGL, the mean of four independent experiments is shown, while the results of a single experiment are shown for the others. Although there was a significant difference in the carbohydrate emptying rate between the control and AGL at GE4 (*p* = 0.039), the difference in values at that point was very small, and the overall tendencies of both were well aligned (Figure 4B). No substantial differences in tendencies were observed for the other thickeners either (Figure A5B). The carbohydrate concentrations of all the enteral formulas decreased proportionately, accompanied by gastric juice inflow and gastric emptying.

## 4. Discussion

Thickeners are commonly used in liquid diets, such as enteral formulas for EN [2,3,4,5] and thickening products for oropharyngeal dysphagia [6,7,8]. The addition of thickeners to enteral formulas is intended to minimize gastric reflux and reduce the burden on caregivers [2,3,4,5,9,10]. Therefore, the impact of thickeners on nutritional value has been largely disregarded. The present study used a semi-dynamic gastric model [17] to evaluate the effects of various thickeners in thickened formulas on the gastric emptying rates of each nutrient, a key factor in nutrient absorption kinetics, and investigated the effects of various thickeners in thickened formulas on the gastric emptying rates of proteins and carbohydrates, which are important factors in nutrient absorption kinetics. The results showed that agar accelerated the gastric emptying rate of proteins, and it was also indicated that this emptying rate could vary depending on the thickener type.

Changes in the gastric emptying rate of proteins were thought to be caused by variations in the degree of aggregate formation during gastric digestion. Sodium caseinate, the protein element in the enteral formulas tested in this study, has been shown to produce aggregation during gastric digestion [12]. In reality, the formula containing no thickeners exhibited aggregation, probably due to sodium caseinate, which corresponded to a drop in pH during gastric digestion. The pyloric diameter limits gastric emptying, and the digesta emptied from the stomach is less than 2 mm in diameter in the human body [20,21]. In our investigation, we used the semi-dynamic gastric model to observe gastric emptying with a pipette having an inner diameter of 2 mm. Hence, the thickener-free formula, which had aggregates that formed during gastric digestion and may have been larger than 2 mm, was not emptied from the stomach and showed a high protein concentration until the latter half of gastric digestion. However, the formula containing agar suppressed aggregate formation depending on the concentration of agar and had a homogeneous appearance. As a result, the gastric emptying of proteins was faster in the agar-containing formula than in the control. Although the mechanism by which agar suppresses aggregate formation is not fully understood in this study, several possibilities can be considered. First, the phenomenon could be explained by the fact that agar, which is mostly composed of the neutral polysaccharide agarose, acts as a near-electrically neutral thickener. Agar is less sensitive to the effects of gastric acid and digestive enzymes; hence, it is used as a model food to demonstrate the influence of physical digestion [24]. Agarose has no functional groups affected by pH. Therefore, it is speculated that the strong network formed by the ordered double helices of agarose [25,26] envelops the casein molecules, making them less susceptible to destruction. This agar–casein network prevents aggregation. Second, the agar molecules may prevent aggregation by blocking interactions between casein molecules that are unrelated to the formation of gel structure. Fontes-Candia et al. [27] found that when a casein solution and agar were mixed without gelation and subjected to in vitro gastric digestion, casein aggregation developed in the absence of a thickener; however, adding agar resulted in the breakdown of these aggregates. This report [27] partially supports our hypothesis. Further research is needed to understand the underlying mechanisms.

In this study, exploratory experiments were conducted with thickeners other than agar to investigate the effect of different types of thickeners on gastric emptying rate. Although the data from these thickeners are based on a single experiment, considering the small standard error of the control and AGL data obtained with the same experimental method, the data could be reliable. In contrast to agar, the addition of carrageenan to the enteral formula resulted in concentration-dependent aggregation. This is most likely the cause of the delayed gastric emptying rate of proteins when compared with that of the control. However, it is unknown how carrageenan stimulates aggregate formation. A possible mechanism is the strong non-covalent interaction between casein and carrageenan. The high electron density of sulfate groups in comparison to carboxyl groups, as well as the negative zeta potential of carrageenan at pH 2–8, have been shown to result in strong hydrogen bonds and electrostatic interactions with casein, which has a positive charge under gastric acid conditions [28,29,30]. Acidified dairy beverages are stabilized with high-methoxyl pectin, which has been shown to suppress casein aggregation. However, a previous study found that the use of low-methoxyl pectin in acidified casein solution caused aggregation due to its high charge density [31]. In other words, extremely strong intermolecular interactions are thought to induce aggregate formation rather than hinder it. Strong electrostatic interactions between the sulfate groups and casein may increase the formation of aggregates. Structural changes in casein and carrageenan may also increase intermolecular interaction, promoting aggregate formation. Guo et al. [32] found that the cations in SGF increase the helical structure of κ-carrageenan, leading to stronger hydrogen bonds and electrostatic interactions with casein. Guo et al. [28] also showed that at pH 3, casein’s random coil turned into rigid structures such as β-sheets and β-turns. This intensified the intermolecular hydrogen bonding between κ-carrageenan and casein through molecular dynamics simulation. Altering the structures of carrageenan and casein to those that are more interactive under gastric conditions may improve their intermolecular interaction and accelerate aggregate formation. Kappa-, iota-, and lambda-carrageenans are commercially available and have one, two, or three sulfate ester groups per dimer [33]. The carrageenan used in this study was mostly ι-carrageenan. Although the number of sulfate groups differs, it is reasonable to assume that ι-carrageenan acts in a similar manner to κ-carrageenan.

Gellan gum in the enteral formula had no impact on the gastric emptying rate of the protein, which was comparable to that of the control. This is probably due to the degree of aggregation. The electrostatic interaction between gellan gum and casein is thought to be weak because carboxyl groups in gellan gum have a lower electron density than sulfate groups. Therefore, it was presumed that gellan gum had no net effect on aggregation, either promoting or suppressing it. The degree of intermolecular interaction is thought to result in aggregation and a gastric emptying rate of proteins similar to that of the control. Guar gum did not affect the gastric emptying rate of the proteins or aggregate formation. Although guar gum is an electronically neutral thickener, similar to agar, it does not have the same effects. This can be attributed to insufficient gelation, as evidenced by the fact that adequate viscosity was not achieved at the concentrations usually used in thickened formulas. Unlike agar, this is thought to result in a similar gastric emptying rate for each nutrient and aggregate formation as the control.

**Figure 5:**
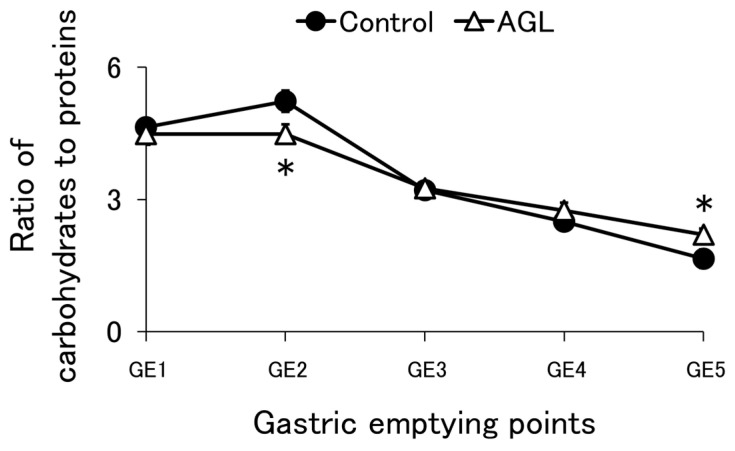
Ratio of carbohydrates to proteins according to the variety of thickeners at each gastric emptying (GE) point. Control = enteral formula containing no thickeners; AGL = enteral formula containing 0.2% agar. Data are shown as mean ± SE (*n* = 4). One asterisk indicates a marginally significant difference at *p* < 0.1 compared to the control (Student’s *t*-test).

**Figure A7:**
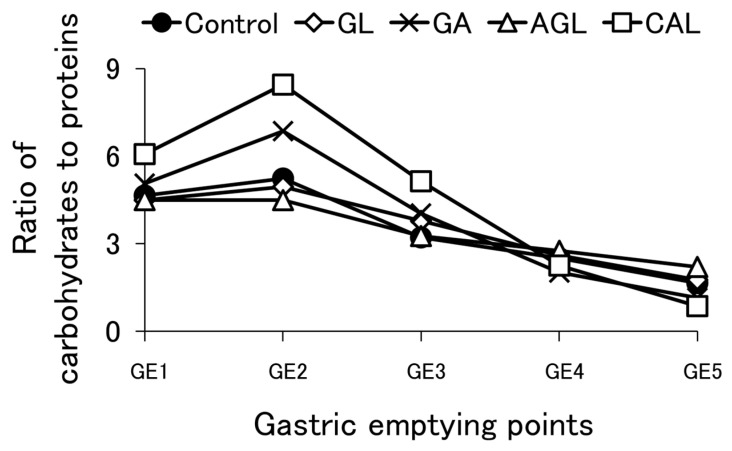
Ratio of carbohydrates to proteins according to the variety of thickeners at each gastric emptying (GE) point. GL = enteral formula containing 0.08% gellan gum; GA = enteral formula containing 0.2% guar gum; and CAL = enteral formula containing 0.08% carrageenan. GL, GA, and CAL each represent the data from a single experiment. Control and AGL are shown for reference, using the same data (the mean of four experiments) as presented in Figure 5.

**Figure A8:**
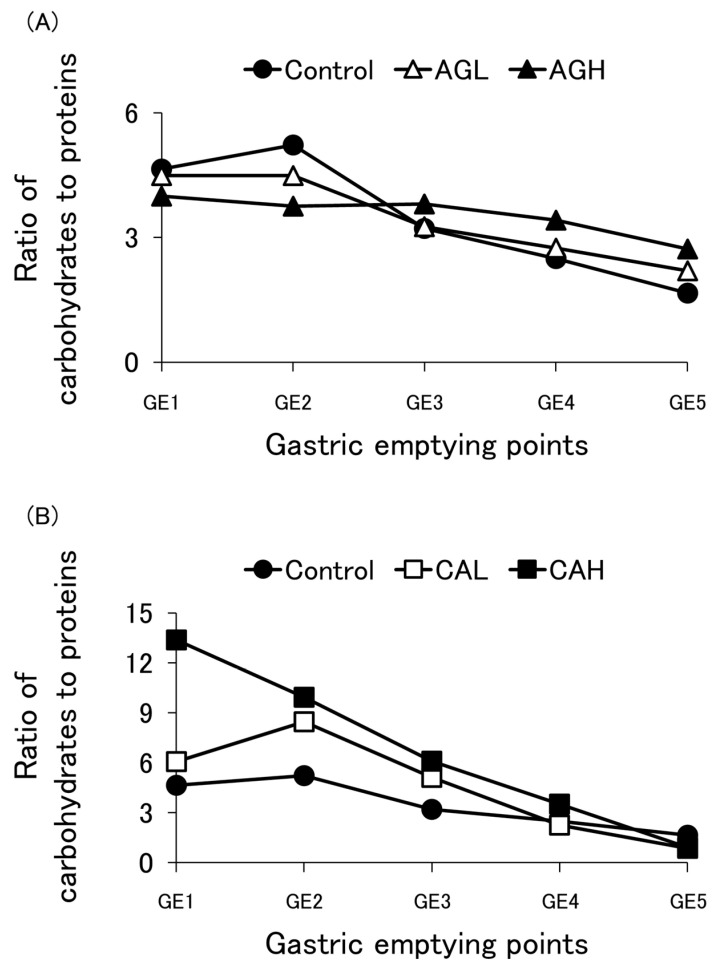
Ratio of carbohydrates to proteins according to the concentration of (**A**) agar and (**B**) carrageenan at each gastric emptying (GE) point. Control = enteral formula containing no thickeners; AGL = enteral formula containing 0.2% agar; AGH = enteral formula containing 0.5% agar; CAL = enteral formula containing 0.08% carrageenan; and CAH = enteral formula containing 0.2% carrageenan. AGH and CAH each represent the data from a single experiment. Control, AGL, and CAL are shown for reference, using the same data as presented in Figure 5 and Figure A7.

Although the gastric emptying rate of proteins changed depending on the type of thickener used, the influence of thickeners on the gastric emptying rate of carbohydrates could not be directly assessed. This implies that the aggregates formed during gastric digestion did not encapsulate carbohydrates. On the other hand, it has been shown that the total number of calories passed on to the duodenum during a given period is determined by the caloric density of the meal through the action of duodenal receptors [34]. If the gastric emptying rate of any nutrient changes, the total amount of caloric gastric emptying over a given period is predicted to remain constant due to feedback control via duodenal receptors, as previously indicated. This causes variations in the gastric emptying rates of other nutrients, resulting in a constant total caloric gastric emptying amount per unit time. For example, if the gastric emptying of proteins accelerates, the emptying of other nutrients may slow down. However, in our study using the semi-dynamic gastric model, it was challenging to collect digesta while keeping the calories constant at each GE; therefore, for practical reasons, the digesta was collected while maintaining a constant volume. To address this limitation, the carbohydrate-to-protein ratio was calculated (Figure 5, Figure A7 and Figure A8A,B). The Atwater factor was 4 kcal/g for both proteins and carbohydrates. It is presumed that the gastric emptying rate of each nutritional component can be examined in a state where the difference in total calories for each GE is corrected by calculating the carbohydrate-to-protein ratio. The control showed a pattern in which the proportion of carbohydrates was high in the early stage of digestion, while the proportion of protein increased in the later stages. However, AGL had low carbohydrate contents during early digestion, showed a trend (*p* = 0.064) to be lower than the control at GE2, and showed a trend (*p* = 0.072) to be higher than the control at GE5 (Figure 5). In exploratory experiments, AGH had low carbohydrate contents during early digestion, like AGL. In contrast, CAL and CAH had higher carbohydrate ratios in the initial phase of digestion. Although this study could not clearly reveal the differences in the gastric emptying of carbohydrates, this method suggests a potential difference. For instance, the addition of agar to the formula may decrease gastric emptying of carbohydrates, while carrageenan accelerates it.

This study found that the gastric emptying rates of proteins and carbohydrates vary depending on the thickener used. However, this study has several limitations. First, the semi-dynamic gastric model was developed using data from healthy individuals. It has been noted that digestive conditions in older adults, such as enzyme levels and gastric pH, differ from those in healthy individuals [35,36,37]. The INFOGEST international network has developed a static in vitro digestion model tailored to the general population of older adults [38]. It is more acceptable to tailor the semi-dynamic gastric model to the subject being evaluated. Second, the semi-dynamic gastric model fails to account for changes in digestion time caused by physical properties. Although caloric density is thought to be the primary factor determining gastric digestion time, physical properties may also play a role [39]. In the semi-dynamic gastric model, digestion time is solely influenced by the caloric density of the test food. Third, the semi-dynamic gastric model cannot completely reproduce peristaltic movement. Although a 3D shaker was used to imitate slow peristaltic movement in the semi-dynamic gastric model, genuine peristaltic movement consists of peristaltic contraction waves that originate in the proximal antrum and proceed to the pylorus [40]. Particle disintegration can be affected by different fluid dynamics and grinding mechanisms. For a more in-depth look at particle disintegration, additional verification using dynamic models, such as a human gastric digestion simulator [24,41], is proposed. Fourth, statistical analysis was only conducted between the control and AGL, for which data from four experiments each were obtained. However, due to the limited number of experiments, the results of this study, with the exception of some data, remained based on observing trends rather than confirming significant differences. Furthermore, for other thickeners, only one exploratory experiment could be conducted for each. The samples used in this study change their characteristics during storage, necessitating the implementation of experiments in a short period of time to obtain appropriate data. However, it was difficult to evaluate a large number of samples simultaneously in this study. We prioritized evaluating samples that had not undergone temporal changes and were in the same state, and we limited the number of samples. On the other hand, to clearly demonstrate significant differences in the experiments with the semi-dynamic gastric model, whether with agar or other thickeners, it is necessary to further increase the number of experiments. Alternatively, it may become possible to demonstrate these differences more clearly by finding a new experimental method. Given these limitations, additional research and clinical trials are needed to improve our findings. Because this study used casein as a protein source, the results may not apply to foods containing other proteins, such as whey. Casein is a protein that is commonly used in food products because of its high nutritional value and ability to withstand sterilization in severe environments with intense heat. Enteral formulas require the long-term, stable preservation of high-density nutrients as well as strong heat sterilization owing to the need for high hygiene. Casein is therefore commonly used as a protein source in enteral formulas. In this study, casein was selected as the protein source. Protein drinks are made from optimal blends of whey protein, which is quickly absorbed, and casein, which is slowly absorbed [42,43], depending on the intended application to maximize assimilation. The same strategies cannot be applied to enteral formulas that use casein as a protein source. Therefore, thickeners can help manage the gastric emptying rate of each nutrient. However, further research is needed to determine the gastric emptying rate of each nutrient in food products made primarily of other proteins as protein sources.

## 5. Conclusions

This study examined the effect of thickeners on the gastric emptying rates of proteins and carbohydrates. The results revealed that agar accelerated the gastric emptying rate of proteins. Furthermore, it was indicated that the gastric emptying rate of proteins and carbohydrates may change depending on the type of thickener used and how well protein aggregation is controlled. We anticipate that the optimal choice of thickeners added to liquid foods based on the patient’s metabolic profile could aid in nutritional management, not only for tube-fed patients but also for those with oropharyngeal dysphagia.

## Data Availability

The datasets generated and/or analyzed in the current study are available from the corresponding author upon reasonable request, due to protection of intellectual property.
